# Time-varying effects of COVID-19 vaccination on symptomatic and asymptomatic infections in a prospective university cohort in the USA

**DOI:** 10.1136/bmjopen-2024-084408

**Published:** 2025-02-22

**Authors:** Lucy Robinson, Anna Feting, Isamu Isozaki, Vicki Seyfert-Morgolis, Mitchell Jay, Edward Kim, Charles Cairns

**Affiliations:** 1 Department of Epidemiology and Biostatistics, Drexel University Dornsife School of Public Health, Philadelphia, Pennsylvania, USA; 2 Department of Computer Science, Drexel University, Philadelphia, Pennsylvania, USA; 3 My Own Med Inc, Bethesda, Maryland, USA; 4 Drexel University, Philadelphia, Pennsylvania, USA; 5 College of Medicine, Drexel University, Philadelphia, PA, USA

**Keywords:** COVID-19, Vaccination, PUBLIC HEALTH

## Abstract

**Abstract:**

**Objectives:**

Despite widespread vaccination programmes and consensus recommendations, the understanding of the durability of COVID-19 vaccination against ensuing infection and transmission at the individual level is incomplete. The objective of this study was to estimate the effects of time-varying covariates including time since vaccination and symptoms on subsequent positive SARS-CoV-2 test results and assess the stability of these effects between March 2020 and April 2022.

**Design:**

Prospective cohort study.

**Setting:**

Urban university in the USA.

**Participants:**

Drexel University students, faculty, and staff (n=15 527) undergoing mandatory COVID-19 symptom tracking, testing and vaccinations.

**Intervention:**

Systematic symptom tracking and SARS-COV-2 testing starting in September 2020 and mandatory COVID-19 vaccination starting in September 2021.

**Main outcomes and measures:**

COVID-19 vaccine effectiveness modified by time since vaccination and symptoms.

**Results:**

Using fit-for-purpose digitally based symptom and vaccine tracking and mandatory comprehensive testing for SARS-CoV-2 infection, we estimate the time-dependent effects of vaccination, symptoms and covariates on the risk of infection with a Cox proportional hazards model based on calendar time scale. We found a strong protective effect of vaccination against symptomatic infection. However, there was strong evidence of a protective effect against infection only in the first 90 days after completed vaccination, and only against symptomatic versus asymptomatic infection. The overall estimated effect of vaccination within 30 days, including asymptomatic infections, was 37.3% (95% CI 26%, 47%). Vaccine effect modification by reported symptoms and time period was estimated, revealing the protective effect of vaccination within 90 days against symptomatic infection that varied from 90% (95% CI 84%, 94%) to 49%(95% CI −77%, 85%) across time periods.

**Conclusions:**

This study is among the first to prospectively capture complete COVID-19 symptom, testing and vaccination data over a multiyear period. Overall effectiveness of the COVID-19 vaccine against subsequent infection, including transmissible asymptomatic infections, is modest and wanes after 90 days. Vaccination policies may need to take these issues into account.

**STRENGTHS AND LIMITATIONS OF THIS STUDY**

- This is a large, prospectively designed cohort study over an extended time period, with complete individual-level data on vaccination history, symptom tracking and collection of both symptomatic and asymptomatic infection events via mandatory weekly PCR testing for SARS-CoV-2.
- Collection of data on asymptomatic SARS-CoV-2 infection events provides insight into vaccine effectiveness against transmission as well as symptoms which is not available in most large surveillance datasets.
- Although the analysis was stratified by age, this university cohort was younger than the larger population and may have had differences with respect to typical symptoms of SARS-CoV-2 infection.

## Introduction

 The reduced risk of death and severe disease from COVID-19 after vaccination is well established.^1^,^3^ Recent data suggest that the protection against SARS-CoV-2 infection is relatively low and wanes rapidly.^4^,^6^ Yet the temporal dynamics of both transmission and symptom protection after vaccination are not completely understood and have been challenging to estimate in the context of an evolving landscape of community infection prevalence, exposure control measures, test availability and dominant virus variants.^7^,^9^ Surveillance data may have incomplete information on individual-level vaccine information and undercount asymptomatic or mild COVID-19 cases, leading to potential biases in studies of long-term vaccine effectiveness. Similarly, the relationship between symptoms and positivity may be complex and vary temporally with season, dominant SARS-CoV-2 variant and prevalence of other respiratory viruses.^7 10 11^ Characterising vaccine effectiveness against both symptoms and transmissibility remains important as policy decisions regarding future COVID-19 vaccinations and guidelines require robust evidence.^10 11^

Previous studies have investigated vaccine effectiveness over time. Yet, many of these studies rely on large, publicly available datasets, which may be incomplete and lack asymptomatic COVID-19 case data.^4 12 13^ As asymptomatic cases appear significant in driving new SARS-CoV-2 infections, especially in Omicron subvariants, understanding the degree to which COVID-19 vaccines are transmission-blocking, and how long this transmission-blocking remains effective is necessary to inform future public health policies and preventative measures, especially for high-risk populations.^14 15^ Entering a new phase of the pandemic, transmission-blocking vaccines would provide the highest level of protection for vulnerable populations. Some studies have used extensive testing and demographic data, but only for limited durations and using less sensitive rapid antigen tests, rather than PCR tests. In this study, we use data from March 2020 to April 2022 on vaccination, SARS-COV-2 PCR testing, and systematically tracked symptoms from a cohort of individuals in the Drexel University community to assess the association between time-varying covariates including vaccination status and symptoms and the risk of a positive test result. This prospective cohort was unique given follow-up throughout different phases of the COVID-19 pandemic, streamlined symptom-tracking with accurate PCR testing and curated vaccine data, including exact dates for the entire vaccination series.

The effect of vaccination on the risk of observed infection and its relationship with symptoms was studied in 15 527 individuals in the cohort. To explore a potentially time-varying effect of vaccination on the risk of infection, as measured by a positive PCR or antigen test, we control for possible confounding by other time-dependent variables using a Cox proportional hazards model based on a calendar time scale. We estimate vaccine effect, defined by time since primary series vaccination or booster, and the modification of this effect by time period and presence of symptoms.

## Methods

### Patient and public involvement

Study subjects were not involved in the design, conduct, reporting or dissemination plans of this research.

### Cohort

All individuals undergoing testing on the Drexel University campus from March 2020 to April 2022, either through the mandatory testing programme or elective testing, or reporting test results through the University app, were eligible for inclusion in the cohort. The cohort consisted of essential employees, faculty and students who attended Drexel University in person. Students lived in on-campus dormitories or off-campus apartments and houses. The cohort was 88.5% students. Demographics of the cohort are described in table 1. The available covariates included date of birth, community member status (student, employee, faculty, other), on-campus housing status, participation in student athletics, gender, reason for testing, known COVID exposure and symptoms (described below).

**Table 1:** Sample characteristics

	Frequency (per cent)
Age at entry	
<18 years	207 (1.4)
18–20	2728 (31.3)
20–22	3888 (25.8)
22–25	2836 (18.8)
25–35	2159 (14.3)
35–50	775 (5.1)
>50	475 (3.2)
Missing	459 (3.1)
Affiliation	
Student	13 734 (88.5)
Employee/other	1793 (11.5)
Gender	
Female	8399 (54.1)
Male	6943 (44.7)
Non-binary/other	185 (1.2)
Mandatory testing	
Yes	7976 (51.3)
No	7551 (48.6)
Total	15 527

Demographic Iinformation for 15 527 subjects in the cohort. .

### SARS-CoV-2 testing information

As part of the mandatory test programme, students and essential staff working and studying on campus were required to have weekly SARS-CoV-2 tests, using the standard method of internasal swabbing and PCR testing performed by trained medical personnel to ensure proper sampling and testing methodologies for maximising sensitivity. A positive, negative or inconclusive result was obtained for each test. Elective testing was also available to all community members. Student athletes were deemed higher risks for SARS-CoV-2 infection and were tested daily.

### Vaccine information

Per Drexel University’s mandated vaccination policy, all community members were required to report vaccine dates and upload images of their vaccination card as well as report vaccine dates at the point of laboratory testing. Vaccine date information was extracted from these data using optical character recognition and manual review to ensure all data regarding type and dates of vaccines was captured in a database. Effective January 2022, all community members present on campus were required to submit evidence of vaccine boosters (Pfizer/BioNTech and Moderna). A small portion of individuals (<2%) were exempted from the vaccine mandate.

### Symptom data collection

In April 2020, a mobile application for tracking COVID-19-associated symptoms (Drexel Health Tracker, My Own Med, Bethesda, Maryland, USA) was developed and deployed to the Apple and Google app stores along with accounts for all Drexel employees, faculty and students. Regardless of in-person or remote status, all members of the Drexel University community were requested to track COVID-19 symptoms to gain an understanding of potential infection rates in the community and surrounding areas of the campus. Importantly, this programme began before the development and Emergency Use Act of SARS-CoV-2 testing, and thus symptoms served as the only known parameter for defining positive infections. Beginning in September 2020, as SARS-CoV-2 PCR testing became widely available,^16^ all members of the Drexel community living or working/studying on campus were required to track symptoms daily using the mobile application. Additionally, during in-person sampling at Drexel sampling centres, all persons were required to report symptoms at the time of sample acquisition. Students, faculty or employees who reported symptoms between weekly scheduled test days were required to obtain a sample and test within 24 hours of symptom appearance.

### Data extraction

Study data were extracted from the Drexel health tracker portal by the data warehouse team (honest broker), entered into a REDCap (Research Electronic Data Capture) database, deidentified and assigned a GUID (Globally Unique Identifier) code for tracking.

#### At-risk cohort

The outcome for each individual in the study is defined as days from entry into the at-risk cohort until positive test result or censoring event. Because membership in the community of individuals involved in the testing programme is dynamic, individuals may enter and leave the cohort. For each subject, we use the first date of SARS-CoV-2 test or symptom tracker use as the time of entry into the at-risk cohort. The time of the first positive test result is used as the event of interest (information from subsequent positive tests is not used), and the time of the last negative test is used as the censoring time if no positive tests are observed.

### Statistical analysis

We used a Cox proportional hazards regression model to estimate adjusted associations between time-varying predictors of interest, including time-since-vaccination status, COVID exposure, reason for testing, and symptoms, and risk of positive SARS-CoV-2 test result. The effect of predictors of interest is adjusted for a dynamic baseline risk of infection which is estimated non-parametrically, and static covariates including gender, community role defined as student, employee, or other, and age at baseline. Each covariate is assessed for violation of the assumption of proportional hazards, that is, whether or not the effect of the covariate on the risk of a positive test remained the same over time. Variables which do not satisfy this assumption were used to define strata of the baseline hazard, allowing us to adjust for effects which vary over time non-parametrically. The hazard for individual *i* in stratum *j* at time *t* is expressed as *h_i_(t*), which depends on a stratum-specific baseline hazard *h_0j_(t*), and effects of static covariates *X*_1_…*X*_p_, and time-varying covariates *Z_1_(t),…, Z_K_:*

$$h_{i}(t)=h_{0j}(t)\;exp\left\{\overset{K}{\underset{k=1}{\Sigma }}\beta_{k}Z_{ik}(t)+\overset{p}{\underset{l=1}{\Sigma }}\gamma_{l}X_{il}\right\}$$

In many applications, the time scale for the Cox model is defined as time on study. Here, however, we use calendar time because we assume there is a common baseline risk of infection based on community prevalence, control measures, and other factors which vary over time. By adjusting for temporal trends, we compare risk between individuals in different vaccination and symptom categories on the same calendar date. To estimate a vaccine effect dependent on time since vaccination, we initially define vaccination status using categories of less than 30, 30-59, 60-89, 90-179, and greater than 180 days since vaccination. The vaccination date is defined as 14 days from the most recent booster or final primary vaccine dose. Vaccination status categories which exhibited no differences were collapsed in a second stage of analysis, in which the interaction between vaccination status and presence of reported symptoms was estimated, as well as how vaccine effectiveness varied over time periods roughly corresponding to epochs of dominant virus variants.

To analyse the variation of vaccine effectiveness over calendar time, we looked at the modification of the vaccine effect and its interaction with symptoms by time period. Time periods were defined a priori based on a combination of the University calendar, which is associated with changes in testing and community exposure as students transition to and from campus living, and changes in dominant viral variant from Delta to Omicron in late 2021. Vaccine effectiveness for symptomatic and asymptomatic infection was estimated within each time period.

## Results

Our study included 15 527 individuals with a median follow-up time of 348 days and a total of 19 437 person-years on study. There were 119 176 test events with an overall test positivity of 23.2%. In the four time periods used to describe time-specific vaccine and covariate effects, test positivity was 24.2% in the pre-September 2021 period, 22.9% in September through November 2021, also 22.9% in December 2021 through January 2022 and 11.6% in February through April 2022. Most of the cohort (96%) received the Pfizer or Moderna mRNA vaccine for their primary vaccination series, and all booster shots were Pfizer or Moderna. Receipt of the Janssen vaccine was reported by 2.5% of the cohort.

Each covariate was tested for violation of the proportional hazards assumption, and we found that age and occupation did not satisfy the assumption. Age was categorised into seven categories, <18 years, 18–20, 20–22, 22–25, 25–35, 35–50 and >50 years, and was used along with occupation to define strata of the baseline hazard. The results were not sensitive to different categorisations of age, and since the sample size and numbers of events observed within each strata were large, categories reflecting qualitative differences within the population (eg, undergraduates vs postgraduates) were chosen.

**Figure 1:**
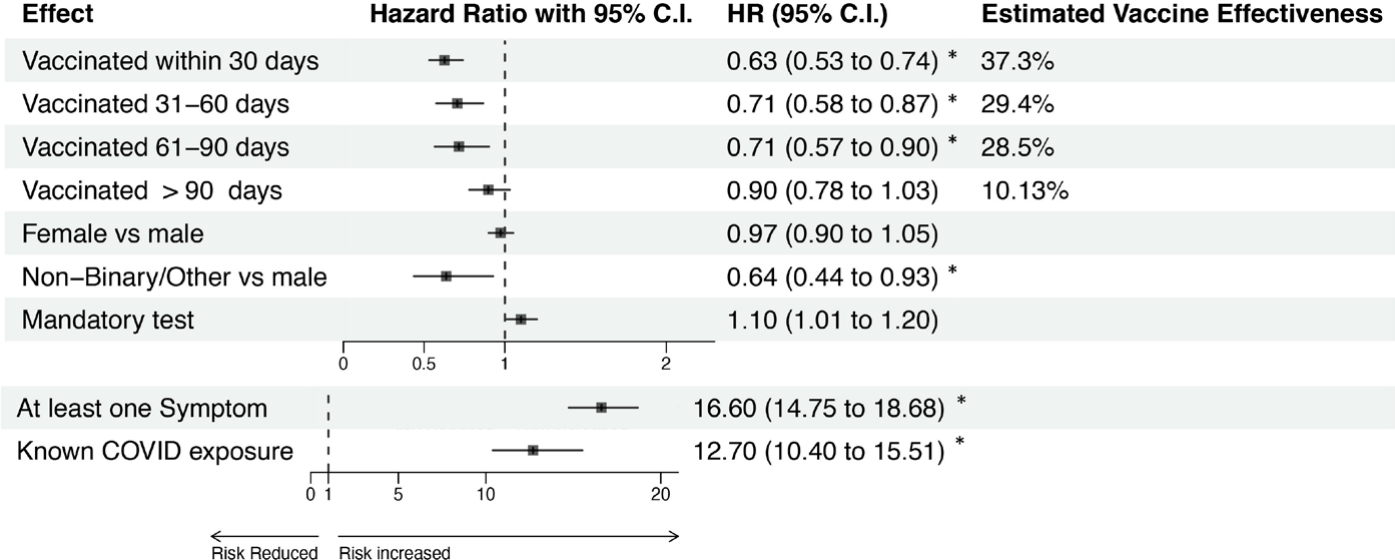
HRs and corresponding 95% confidence limits for vaccination and covariate effects. Results from the proportional hazards regression model predicting the hazard of COVID-19 infection, as measured by a positive PCR or antigen test, from time-since-vaccination status and covariates. Each variable was tested for violation of the proportional hazards assumption; age and occupation (student vs employee/other) did not satisfy the assumption, and thus were used to define strata of the baseline hazard. Time since vaccination is defined relative to 14 days after the final dose of the primary series, or 14 days since the most recent booster. Vaccine effectiveness is 1−HR. The HRs associated with symptoms and exposure (bottom two rows) are plotted using a different scale than the other effects for ease of visual interpretation. Estimated HRs whose 95% confidence limits do not contain 1 are indcated by ‘*’.

**Figure 2:**
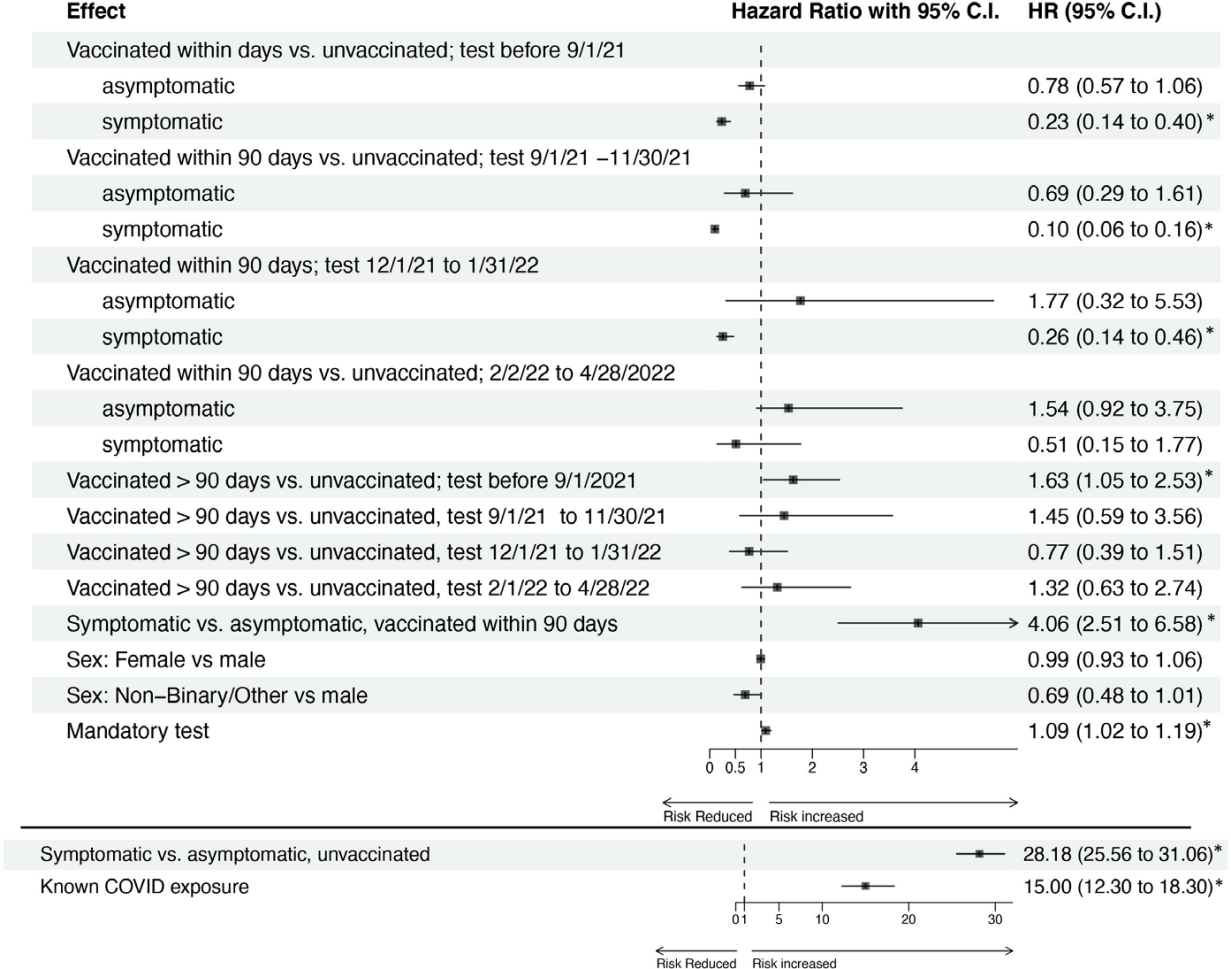
HR and corresponding 95% confidence limits for vaccination effects modified by symptomaticity and time period, and covariate effects. Results from the proportional hazards regression model predicting the hazard of COVID-19 infection, as measured by a positive PCR or antigen test, from time-since-vaccination status modified by time period and symptom status, adjusted for covariates. Age and occupation (student vs employee) were used to define strata of the baseline hazard. Time since vaccination is defined relative to 14 days after the final dose of the primary series, or 14 days since the most recent booster. Vaccine effectiveness is 1−HR. The HRs associated with symptoms and exposure (bottom two rows) are plotted using different visual scales than the other effects for ease of visual interpretation. Estimated HRs whose 95% confidence limits do not contain 1 are indcated by ‘*’.

The effect of vaccination on the risk of positive SARS-CoV-2 test was found to be complex, differing for symptomatic versus asymptomatic presentation, and with a magnitude that depends on time since vaccination and time period. Figure 1 shows estimates of overall vaccine effectiveness by categories of time since vaccination and adjusting for presence of at least one symptom, known COVID-19 exposure, gender and high-risk mandatory testing status. After observing no difference between the categories of 90–180 days and more than 180 days, we collapsed them into a single category. We found strong evidence of overall vaccine effectiveness within 90 days of vaccination, with estimated vaccine effectiveness over the less than 30 days, 30–59 days and 60–90 days of full vaccination categories of 37.3% (95% CI 26%, 47%), 29.4% (95% CI 13%, 42%) and 28.5% (95% CI 10%, 43%), respectively. No evidence of vaccine effectiveness after 90 days was observed, with an estimated vaccine effect of 7.1% (95% CI −6.0%, 18.6%). Results for vaccine effectiveness by time period and symptom status are shown in figure 2. With modification by time period and reported symptoms, estimated vaccine effectiveness within 90 days of full vaccination for symptomatic infections from 1 September 2021 to 30 November 2021 was 90% (95% CI 84%, 94%) as compared with 31% (95% CI −61%, 71%) for asymptomatic infections over the same time period. In the pre-September 2021 time period, estimated vaccine effectiveness within 90 days of full vaccination for symptomatic infections was 77% (95% CI 60%, 84%) and 22% (95% CI −6%, 43%) for asymptomatic infections. In the 1 December 2021 to 31 January 2022 time period, estimated vaccine effectiveness within 90 days of full vaccination for symptomatic infections was 74% (95% CI −54%, 8%) and with a highly uncertain estimated effect of −77% (95% CI −453%, 68%) for asymptomatic infections. Estimated vaccine effectiveness was lower with wide CIs in 1 February 2022–28 April 2022 time period, with estimates of 49% (95% CI −77%, 85%) and −54% (95% CI −275%, 8%) for symptomatic and asymptomatic infections, respectively.

Individual symptoms reported within 14 days prior to the SARS-CoV-2 test were analysed as predictors of positive results. We found that body aches, chills, fatigue, cough, headache, sore throat, vomiting and diarrhoea, fever, and loss of taste or smell are all associated with increases in the hazard of a positive test result, after controlling for other symptoms, vaccination status and covariates.

## Discussion

This is one of the first prospective studies to estimate vaccine effectiveness against both asymptomatic and symptomatic SARS-CoV-2 infection in a prospectively studied cohort which was regularly PCR-tested and tracked for symptoms. We studied the waning of effectiveness over individual-level time since vaccination as well as the consistency of vaccine effectiveness across time periods spanning the first 2 years of the pandemic for both symptomatic and all detected infections. In contrast to the relatively high protection against severe, symptomatic infection that has been widely reported,^4^ we observed moderate and time-limited protection against less severe and asymptomatic infection, consistent with recent findings.^5^ We find that the effect of vaccination on infection wanes after approximately 90 days and differs substantially between symptomatic and asymptomatic presentation. Based on analyses stratified by time period, until early 2022, differences in vaccine effectiveness over time appear to be explained primarily by waning of effectiveness as time since vaccine increases for individuals, rather than changes in virus tied to calendar time. We observed a decline in vaccine effectiveness towards the end of our follow-up period, beginning early 2022, which corresponds to the emergence of the Omicron variant as the dominant virus strain.

The study design had several strengths. Our observed follow-up period from September 2020 to April 2022 spanned highly dynamic phases of the pandemic, in which the overall vaccination status of the cohort shifted from completely unvaccinated to nearly completely vaccinated over an extended period, allowing for comparison between unvaccinated and vaccinated individuals at a range of time points. This relatively long follow-up period allows us to assess the stability of estimated vaccine effectiveness over time as new virus variants emerged and became dominant, and community infection control measures changed. The regular testing of individuals is also a key feature of the study design, which allows us to detect infections in individuals with mild or no symptoms, or individuals who may be disinclined to test. Particularly in a young cohort, the detection of mildly symptomatic or asymptomatic infections provides key insights into the potential effectiveness of vaccines against transmission as well as symptoms. Studies of virus shedding among vaccinated individuals^17^ confirm the importance of transmission by asymptomatic infected individuals in COVID-19 spread.

Our study had some limitations with respect to potential sources of protection from infection not described by measured vaccination status, including possibly incomplete information on previous infection prior to entry into the cohort, which may have provided some protection against reinfection for unvaccinated individuals. However, the study began early in the pandemic, and given the vaccine mandate, we believe the potential association between vaccination and prior infection will be weak, reducing the possibility of confounding in the estimate of vaccine effectiveness. Additionally, the time of vaccination was defined as 14 days following the last dose of the primary series or booster. The potential protection provided by the initial dose of a two-dose primary vaccine series, or in the 14 days following the last dose, was not reflected in our calculation of vaccine effectiveness. Symptoms were self-reported via a combination of a symptom-tracking app and report at point of testing. Recorded data from the 14 days prior to testing was used to define symptom status, but irregular use of the tracking app for individuals not regularly on campus may have introduced some measurement error.

Our estimated vaccine effectiveness against infection is consistent with recent reports,^4 5^ but not with retrospective studies from the earlier stages of the pandemic.^18^ Of note, these earlier studies did not include systematic testing among asymptomatic individuals, which accounts for a significant number of SARS-CoV-2 infections.^19^ Vaccine effectiveness depends on viral variants,^8^ and the extended follow-up period of our study allowed for examination of vaccine effectiveness over time periods characterised by the dominance of Alpha and Delta variants, and the emergence of the Omicron variant. Our findings on vaccine effectiveness are also consistent with those found during both the Delta^4 20^ and Omicron variant phases.^5 21^

In addition to assessing the stability of the vaccine effect over calendar time, we explored the temporal dynamics of vaccine effectiveness within individuals as a function of time since vaccination. We found a marked time-sensitivity to the protection of vaccination against infection, with a cut-off of approximately 90 days, consistent with studies on antibody responses after vaccination.^22^ This relatively short duration of protection against infection is consistent with that reported for viral load reduction effectiveness.^13^ The waning of vaccine effectiveness over time observed in our cohort has consistently been observed in other populations. A meta-analysis of vaccine effectiveness studies^23^ included estimation of a decay function of vaccine effectiveness over time for both symptomatic and all laboratory-confirmed infection for the Omicron and Delta variants. Their results are broadly consistent with ours for initial effectiveness, with pooled estimates of vaccine effectiveness against symptomatic infection of 79.6% at 1 month after completion of the primary vaccination cycle, 58.5% at 6 months and 49.7% at 9 months. Other meta-analyses^1^ of waning vaccine effectiveness found VE decreased on average by 24.9 percentage points between 1 month and 6 months after the final vaccine dose. The longer-term protection efficacy rate against infection found in this study (28%) is consistent with the longer-term neutralisation rates reported after vaccination (~25%).^6^ More recent studies^24^,^26^ of the effectiveness of bivalent boosters report similarly moderate and time-limited protection against infection.

The tracking of symptoms in the days prior to and at the point of testing allowed us to understand the association between individual symptoms and SARS-CoV-2 test positivity under regular testing and in real-world conditions. Thus, a risk-stratification approach that uses symptoms could facilitate targeted testing programmes where testing resources are limited or high-risk populations need to be identified for frequent testing.

The implications of these findings are relevant to COVID-19 vaccination policies. For example, both the FDA and CDC recently recommended annual COVID-19 boosters.^14 15^ Our findings suggest that because protection from infection, including asymptomatic infection, is incomplete and wanes after approximately 90 days, the timing of an annual vaccination must be carefully considered to maximise effectiveness.

## Data Availability

Data are available on reasonable request.
