# Supplementary material for: Time-varying effects of COVID-19 vaccination on symptomatic and asymptomatic infections in a prospective university cohort in the USA
Source: BMJ Open. 2025 Feb 22;15(2):e084408. doi: 10.1136/bmjopen-2024-084408 (PMC11848656; doi:10.1136/bmjopen-2024-084408)
Supplement: Supplementary file 1 [file bmjopen-2024-084408.reviewer_comments.pdf]

PEER REVIEW HISTORY

BMJ Open publishes all reviews undertaken for accepted manuscripts. Reviewers are asked to complete a checklist review form and are provided with free text boxes to elaborate on their assessment. These free text comments are reproduced below.

ARTICLE DETAILS

Title (Provisional)

Time-varying effects of COVID-19 Vaccination on Symptomatic and Asymptomatic Infections in a Prospective University Cohort in the United States

Authors

Robinson, Lucy; Feting, Anna; Isozaki, Isamu; Seyfert-Morgolis, Vicki; Jay, Mitchell; Kim, Edward; Cairns, Charles

---

VERSION 1 - REVIEW

**Reviewer**	**1**
**Name**	**Kadhim, Kadhim Fadhil**
**Affiliation**	**University of Basrah, Department of Biology**
**Date**	**08-Sep-2023**
**COI**	**None**

Comments to the Author:

Please take the notes below into consideration and adopt them as much as possible

1. Reference No. 9 and 10 are missing in the text of the research and is present in the list of references.
2. Reference No. 16 is missing from the list of references and is present in the research text.
3. On page 12, line 10, I mentioned that the results agree with some but not all. This is a general statement and applies to most of the results of studies. explain with references the agree and disagree.
4. On page 7, line 43. Do you ensure that all people report symptoms correctly via the app? What is the possible percentage of error (-+) that must at least be taken into account in the results?
5. On page 8, line 27. Need to ethical approval by No. and date.
6. On page 7, line 15-17 (Student athletes were deemed higher risks for COVID-19 infection and were tested daily). Did you rely on that in your work? Do you have references to support your accreditation?
7. Statistical marker must be included in the tables, if any.

8. The discussion is very short and weak and needs more insightful discussion and supporting references.

Reviewer **2**

Name **Knol, Mirjam**

Affiliation **Rijksinstituut voor Volksgezondheid en Milieu, Infectious Disease Control**

Date **25-Sep-2023**

COI **None.**

Comments to the Author:
The authors tried to assess vaccine effectiveness, and specifically it duration, on symptomatic and asymptomatic infection. At some points I do not understand, or agree with, the methodology chosen by the authors.

Major comments:
1. From the abstract it seems that vaccination is mandatory at the university. To calculate VE also unvaccinated participants are needed, so if vaccination is mandatory how can VE be calculated. In the methods it is stated that <2% was not vaccinated due to an exempt. Please report the exact percentage and number of unvaccinated participants. In any case, this is a very small number. Also, it is important to state during which time period participants were vaccinated, as the VE estimate will be mainly driven by the contrast between vaccinated and unvaccinated during this time window.
2. It is unclear whether the VE presented was for primary or booster vaccination. Or both combined?
3. In the results, number of infections and persontime should be presented.
4. The authors adjust for presence of symptoms in their analysis. This is not correct. presence of symptoms is not a confounder in the association between vaccination and infection. It is actually a result of infection.
5. The same holds for adjustment of known COVID-19 exposure. How is this a confounder in the view of the authors?
6. Describe in the results section what was found for other time periods and covariates instead of merely referring to Figure 2.
7. In the discussion, the authors state that they saw minimal change in VE over the two year pandemic period. On which observation was this based? Based on the results in Figure 1, the VE against symptomatic infection was clearly lower in the latest period compared with previous periods. Also, the literature on VE is very consistent in showing lower VE estimated against Omicron infection compared with prior variants.
8. The discussion is very short and not describing any limitations of the study.

9. Please elaborate on the statement that the findings of this study suggest that annual COVID-19 boosters may not protect against infection transmission. As the study did not directly assess the effect of vaccination on transmission.
10. In Table 1 it appears that about 50% of the participants were subject to mandatory testing. From the paper it seemed that all staff and students had to test. Please explain.

Minor comments:
1. SARS-CoV-2 is the virus causing the infection and this infection can cause the disease COVID-19. So please use this terminology correctly, e.g. it is 'SARS-CoV-2 infection' SARS-CoV-2 test'.
2. Reference 16 is missing in the reference list.
3. In Figure 2 the point estimates and confidence interval lines are missing.

**Reviewer**	**3**
**Name**	**Tan, Suriyani**
**Affiliation**	**Universitas Trisakti**
**Date**	**25-Sep-2023**
**COI**	**None.**

Comments to the Author:
1. In Introduction, there is citation 16, but there is no reference no 16 in the references list.
2. Citation 9 and 10 can not be found in the text, but there are reference 9 and 10 in the references list
3. In results part, the authors do not explain about the age categorization, why should the age categorized into 7 categories
4. There should be no intervention for cohort study
5. This manuscript stated that they use prospective cohort study, but there is no information of the groups classification of the study. In cohort study, there should be 2 groups (the exposed and non-exposed)

VERSION 1 - AUTHOR RESPONSE

Reviewer Reports:

Reviewer: 1
Dr. Kadhim Fadhil Kadhim, University of Basrah, National university of sciences and technology, iraq

Comments to the Author:
Please take the notes below into consideration and adopt them as much as possible
1. Reference No. 9 and 10 are missing in the text of the research and is present in the list of references.

We thank the reviewer for catching this error, and have corrected the list of references. Indeed, we have updated the references with new literature.

2. Reference No. 16 is missing from the list of references and is present in the research text.

Again, we thank the reviewer for catching this error, and have added the missing reference.

3. On page 12, line 10, I mentioned that the results agree with some but not all. This is a general statement and applies to most of the results of studies. explain with references the agree and disagree.

We appreciate the suggestion to clarify how the results of our study relate to previous findings, and have modified the first and third paragraphs of the discussion section to provide more detailed comparisons to other studies. Specifically, we have added new literature references to contrast the findings of studies published earlier in the pandemic from more recent literature, especially regarding the relatively low rates of protection against infection after vaccination.

4. On page 7, line 43. Do you ensure that all people report symptoms correctly via the app?

The application went through multiple pre-release versions with no errors noted in symptom reporting. In addition to collection of symptoms on the app, data on symptoms are collected at the point of testing.

5. On page 8, line 27. Need to ethical approval by No. and date.

The study was approved by the Drexel Institutional Review Board (protocol 2106008628) approved as an exempt protocol on July 2, 2021. This information has been added to the paper.

6. On page 7, line 15-17 (Student athletes were deemed higher risks for COVID-19 infection and were tested daily). Did you rely on that in your work? Do you have references to support your accreditation? T

his presumption was based on the high degree of daily, often unmasked contact within groups of athletes during the athletic season, in addition to the requirement by the athletic conference (CAA) and National Collegiate Athletic Association for basketball practice and participation.

7. Statistical marker must be included in the tables, if any.

We have added markers for statistical significance to the figures.

8. The discussion is very short and weak and needs more insightful discussion and supporting references.

We have expanded the discussion to include recent published articles on infection protection after vaccination, how our results fit into the current body of knowledge on the temporal stability of vaccine effectiveness and implications of these findings.

Reviewer: 2
Dr. Mirjam Knol, Rijksinstituut voor Volksgezondheid en Milieu

Comments to the Author:
The authors tried to assess vaccine effectiveness, and specifically it duration, on symptomatic and asymptomatic infection. At some points I do not understand, or agree with, the methodology chosen by the authors.

Major comments:
1. From the abstract it seems that vaccination is mandatory at the university. To calculate VE also unvaccinated participants are needed, so if vaccination is mandatory how can VE be calculated. In the methods it is stated that <2% was not vaccinated due to an exempt. Please report the exact percentage and number of unvaccinated participants. In any case, this is a very small number. Also, it is important to state during which time period participants were vaccinated, as the VE estimate will be mainly driven by the contrast between vaccinated and unvaccinated during this time window.

The percentage of unvaccinated at-risk participants varied continually over the course of the study, and most participants were unvaccinated during the initial period of follow-up. Under this study design, most subjects are observed both before and after their date of full vaccination. Thus, they contribute information in both their unvaccinated and vaccinated states, and their

vaccine status is updated over the course of follow-up. As the reviewer states, the estimate of vaccine effectiveness is based on a comparison of risk between vaccinated and unvaccinated individuals after controlling for a time-specific baseline hazard.

2. It is unclear whether the VE presented was for primary or booster vaccination. Or both combined?

As stated in the methods section and figure captions, vaccination date is defined as 14 days from most recent booster or final primary vaccine dose, whichever is most recent.

3. In the results, number of infections and person time should be presented.

We appreciate this suggestion and have added these summary data to the results.

4. The authors adjust for presence of symptoms in their analysis. This is not correct. presence of symptoms is not a confounder in the association between vaccination and infection. It is actually a result of infection.

The reviewer is correct that we do not consider symptoms as a confounder in the relationship between vaccine status and infection. In the analysis presented in table 2, we include symptoms as an effect modifier, to estimate the effectiveness of vaccine against both symptomatic and asymptomatic infection. In the results for overall vaccine effectiveness presented in table 1, we present symptoms as a covariate to provide information about the increase in test positivity associated with symptom status.

5. The same holds for adjustment of known COVID-19 exposure. How is this a confounder in the view of the authors?

The reviewer is correct that like symptoms, we do not consider exposure as a confounder in the relationship between vaccine status and infection. In the results for overall vaccine effectiveness presented in table 1, we present exposure as a covariate to provide information about the increase in test positivity associated with exposure status.

6. Describe in the results section what was found for other time periods and covariates instead of merely referring to Figure 2.

We appreciate the suggestion to expand the results reported in the text, and have added description of the results for estimated vaccine effectiveness as modified by time period and symptom status.

7. In the discussion, the authors state that they saw minimal change in VE over the two year pandemic period. On which observation was this based? Based on the results in Figure 1, the VE against symptomatic infection was clearly lower in the latest period compared with previous periods. Also, the literature on VE is very consistent in showing lower VE estimated against Omicron infection compared with prior variants.

We thank the reviewer for pointing out this ambiguity, and have updated the discussion to reflect the decrease in the estimated VE observed post February 2022.

8. The discussion is very short and not describing any limitations of the study.

We have expanded the discussion of the findings in the context of recent literature on vaccine effectiveness and added some commentary on limitations.

9. Please elaborate on the statement that the findings of this study suggest that annual COVID-19 boosters may not protect against infection transmission. As the study did not directly assess the effect of vaccination on transmission.

The reviewer is correct that we did not measure transmission in this study. Our comment on the short duration of vaccine effectiveness and the weakness of the vaccine effect against asymptomatic infection, which may be transmissible, has been clarified.

10. In Table 1 it appears that about 50% of the participants were subject to mandatory testing. From the paper it seemed that all staff and students had to test. Please explain.

The measure of mandatory testing refers to the test rather than the individual. A subject undergoing mandatory testing may also appear for unscheduled testing.

Minor comments:
1. SARS-CoV-2 is the virus causing the infection and this infection can cause the disease COVID-19. So please use this terminology correctly, e.g. it is 'SARS-CoV-2 infection' SARS-CoV-2 test'.

The descriptions of test and infection have been corrected to SARS-CoV-2.

2. Reference 16 is missing in the reference list.

We thank the reviewer for catching this error, and have corrected the list of references. Indeed, we have updated the references with new literature.

3. In Figure 2 the point estimates and confidence interval lines are missing.

We have added markers for statistical significance to the figure.

Reviewer: 3
Dr. Suriyani Tan, Universitas Trisakti

Comments to the Author:
1. In Introduction, there is citation 16, but there is no reference no 16 in the references list.

We thank the reviewer for catching this error, and have corrected the list of references. Indeed, we have updated the references with new literature.

2. Citation 9 and 10 can not be found in the text, but there are reference 9 and 10 in the references list

We thank the reviewer for catching this error, and have corrected the list of references. Indeed, we have updated the references with new literature.
.
3. In results part, the authors do not explain about the age categorization, why should the age categorized into 7 categories.

The age categorization for stratification was based on balancing the objectives of describing potential differences in baseline hazard between age groups, for example undergraduates, post-undergraduates, and older adults, and achieving adequate numbers of infection across time periods for each group. A sensitivity analysis showed very similar results for 2, 4, or 7 age categories. A statement to this effect has been added to the results section.

4. There should be no intervention for cohort study.

This is an observational study, in which the time-dependent effect of exposure to vaccine is studied.

5. This manuscript stated that they use prospective cohort study, but there is no information of the groups classification of the study. In cohort study, there should be 2 groups (the exposed and non-exposed).

In this study, the time-dependent effect of exposure to vaccine on risk of positive test for SARS-CoV-2 infection is estimated. Under this study design, most subjects are part of the at-risk cohort both before and after their date of full vaccination. Thus, they contribute information in both their unvaccinated and vaccinated states, or exposed and unexposed states, and their vaccine status is updated over the course of follow-up.

Reviewer: 1
Competing interests of Reviewer: I have no competing interests

Reviewer: 2
Competing interests of Reviewer: I have no competing interests.

Reviewer: 3
Competing interests of Reviewer: None

If you elected during submission to send your article on to another journal the article will be transferred in 5 working days. If you intend to rebut this decision please notify us before then.
The journal(s) (if any) you have selected at submission are:
If you want to speed up or stop this onward transmission please email the editorial office: info.bmjopen@bmj.com

Reply

Reply all

Forward

VERSION 2 - REVIEW

**Reviewer**	**1**
**Name**	**Tan, Suriyani**
**Affiliation**	**Universitas Trisakti**
**Date**	**29-Jan-2024**
**COI**	**None**

-

**Reviewer**	**2**
**Name**	**Lu, Yihan**
**Affiliation**	**Fudan University School of Public Health, Department of Epidemiology**
**Date**	**04-Mar-2024**
**COI**	**None.**

The study provided the evidence to support the VE in the early pandemic.

1, The authors may add more to illustrate the SARS-CoV-2 strains in the early pandemic, such as prototype and variants, in the discussion. These strains shared different epidemic scenarios, particularly prototype strains, alpha, and beta vs delta and omicron variants.

2, In the study, approximately 49% of the respondents did not receive mandatory testing. So I wonder if the findings might be biased due to lack of laboratory confirmation. A further sub-group analysis would facilitate accurately measuring and confirming the VE.

**Reviewer**	**3**
**Name**	**Chong, Ka**
**Affiliation**	**The Chinese University of Hong Kong**
**Date**	**08-Jun-2024**
**COI**	**None**

This is a revised paper titled "Time-varying effects of COVID-19 Vaccination on Symptomatic and Asymptomatic Infections in a Prospective University Cohort: A Real-World Effectiveness Study". Although the authors have addressed some comments from the reviewers, there are still some major concerns.

1. Most of the real-world study used population-level data as we would like to generalize the findings in a large extend. This study only used the data from an University that should not use "real-world" this common term. Please remove all these descriptions in the paper.

2. In the objective of abstract, please remove "We estimate the effects of time

-varying covariates..." as they are just for being adjusted in this observational study.

3. Please replace all "Time-varying effects of COVID-19 Vaccination" to "Waning effects of..." as the monotonic trend is very clear that the vaccine effect waned by time.

4. There are many studies thta have examined the waning effect of COVID-19 vaccine. Please cite enough key papers in the background and discussion.

5. The last paragraph of the introdcution should highlight the existing research gap and point out the study hypothesis (aim), instead of writing a summary.

6. Vaccine information: "Effective January 2022..." ???

7. The information of "Cohort" section is not clear enough. Please completely revise this paragraph and let us know what is the study population (e.g. eligibility criteria, population background, subject recruitment, follow up date...)

8. "SARS-COV-2 testing information": I cannot see what information have been obtained.

9. What baseline characteristics have been collected? Please describe in Methods

10. Please specific clear what are the time-varying and fixed covariates in this study. "time

-since-vaccination status", what is it?

11. The manuscript requires a comprehensive English editing including an editing of sentence and paragraph structure.

**Reviewer**	**4**
**Name**	**Prakken, Berent**
**Affiliation**	**UMC Utrecht Faculty of Medicine**
**Date**	**19-Jun-2024**
**COI**	**I have no competing interest**

In this prospective cohort study Robinson et al systematically tracked symptoms from a cohort of individuals at Drexel University March 2020 to April 2022, so at the heart of the COVID pandemic. They used these data on vaccination, SARS-COV-2 PCR testing to assess the association between among others vaccination status, symptoms and the risk of a positive test result. This was done in a prospective cohort 15.527 University students, faculty, and staff (n=15,527) at Drexel that underwent mandatory COVID-19 symptom tracking, testing and vaccinations.

I think that there are various important novelties in this study that adds to already extensive wealth of data on the dynamics of the COVID pandemic. First of all the large prospective cohort albeit with a relatively young population. Next the use of a mobile device to track the use of an application on mobile devices for tracking the symptoms.

This is as far as I know a quite unique, real-world study that gives important new insights in the dynamics of the COVID pandemic in a well-described cohort. It provides a fascinating picture of the first important two years of the COVID pandemic and the efficacy of vaccination on both symptomatic and asymptomatic infections. One of the most relevant findings is that the overall effect of vaccination on asymptomatic infections was quite low (estimated overall effect on infections within 90 days around 37,3%). Their overall conclusion, namely that the differences in vaccine effectiveness over time appear to be driven primarily by evolution of individual level time-since vaccine status and not so much by changes in the virus, is in my opinion very significant. Given the way the study was structured, the use of the mobile app and the impressive amount of data makes this a robust study that in my opinion yields important new insights in the dynamics of the COVID pandemic. It would even be advisable to add a commentary or editorial to put this study in a broader perspective.

Minor remarks: some small typo's in the manuscript (eg requited instead of required)

VERSION 2 - AUTHOR RESPONSE

Dear Editors and Reviewers,

Thank you very much for your valuable suggestions on our manuscript with the updated title "Time-varying effects of COVID-19 Vaccination on Symptomatic and Asymptomatic Infections in a Prospective University Cohort in the United States". We appreciate your time and effort providing feedback to improve our manuscript. We have incorporated the reviewer's suggestions. Please see below for a point-by-point response to the reviewer's comments.

Sincerely,

Lucy Robinson

Reviewer Reports:

Reviewer: 1
Dr. Suriyani Tan, Universitas Trisakti

Comments to the Author:
-

Reviewer: 2
Dr. Yihan Lu, Fudan University School of Public Health

Comments to the Author:
The study provided the evidence to support the VE in the early pandemic.

1, The authors may add more to illustrate the SARS-CoV-2 strains in the early pandemic, such as prototype and variants, in the discussion. These strains shared different epidemic scenarios, particularly prototype strains, alpha, and beta vs delta and omicron variants.

We have added the following paragraph to the end of the methods section to clarify how vaccine effectiveness was estimated within time periods characterized by Delta and Omicron variant dominance.

> To analyze the variation of vaccine effectiveness over calendar time, we looked at the modification of the vaccine effect and its interaction with symptoms by time period. Time periods were defined *a priori* based on a combination of the University calendar, which is associated with changes in testing and community exposure as students transition to and from campus living, and changes in dominant viral variant from Delta to Omicron in late 2021. Vaccine effectiveness for symptomatic and asymptomatic infection was estimated within each time period.

2, In the study, approximately 49% of the respondents did not receive mandatory testing. So I wonder if the findings might be biased due to lack of laboratory confirmation. A further sub-group analysis would facilitate accurately measuring and confirming the VE.

The reviewer raises an interesting point which we have now included in the discussion section. While mandatory testing status could potentially be associated with probability of detected infection, it is adjusted for as a potential confounder in the analysis of vaccine effectiveness, and since all individuals in the community were subject to the same vaccine mandate, we did not find differences in vaccination rates between individuals subject to and not subject to mandatory testing.

Reviewer: 3
Dr. Ka Chong, The Chinese University of Hong Kong

Comments to the Author:
This is a revised paper titled "Time-varying effects of COVID-19 Vaccination on Symptomatic and Asymptomatic Infections in a Prospective University Cohort: A Real-World Effectiveness Study". Although the authors have addressed some comments from the reviewers, there are still some major concerns.

1. Most of the real-world study used population-level data as we would like to generalize the findings in a large extend. This study only used the data from an University that should not use "real-world" this common term. Please remove all these descriptions in the paper.

We have removed 'real-world' from the title, and clarified in the text that the context is a University.

2. In the objective of abstract, please remove "We estimate the effects of time -varying covariates..." as they are just for being adjusted in this observational study.

We thank the reviewer for the opportunity to clarify this point. In addition to time-varying vaccination status, symptoms, exposure, and reason for testing are also time-varying. (For a given individual, a single testing event can be mandatory or not.) Estimates of time varying covariates can be found in tables 2 and 3.

3. Please replace all "Time-varying effects of COVID-19 Vaccination" to "Waning effects of..." as the monotonic trend is very clear that the vaccine effect waned by time.

We consider both the variation of vaccine effect since time of vaccination within individuals, which is decreasing, and the variation of vaccine effect over calendar time.

4. There are many studies thta have examined the waning effect of COVID-19 vaccine. Please cite enough key papers in the background and discussion.

We have expanded the discussion section to include more literature on waning vaccine effects

5. The last paragraph of the introdcution should highlight the existing research gap and point out the study hypothesis (aim), instead of writing a summary.

We have removed the summary of results from the introduction.

6. Vaccine information: "Effective January 2022..." ???

We have rewritten this sentence for clarity.

7. The information of "Cohort" section is not clear enough. Please completely revise this paragraph and let us know what is the study population (e.g. eligibility criteria, population background, subject recruitment, follow up date...)

The subsection has been revised as suggested and now reads as follows

> All individuals undergoing testing on the Drexel University campus from March 2020 to April 2022, either though the mandatory testing program or elective testing, or reporting test results through the University app, were eligible for inclusion in the cohort. The cohort consisted of essential employees, faculty, and students who

attended Drexel University in person. Students lived in on-campus dormitories and off-campus apartments and houses. The cohort was 88.5% students. Demographics of the cohort are described in Table 1. The available covariates included date of birth, community member status (student, employee, faculty, other), on-campus housing status, participation in student athletics, gender, reason for testing, known covid exposure, and symptoms (described below).

8. "SARS-COV-2 testing information": I cannot see what information have been obtained.

The following sentence has been added to clarify this point

A positive, negative, or inconclusive result was obtained for each test.

9. What baseline characteristics have been collected? Please describe in Methods
We have added the following to the cohort subsection of the methods section.

The available covariates included date of birth, community member status (student, employee, faculty, other), on-campus housing status, participation in student athletics, gender, reason for testing, known covid exposure, and symptoms (described below).

10. Please specific clear what are the time-varying and fixed covariates in this study. "time -since-vaccination status", what is it?

To clarify this point, we added a description of the static covariates to the end of the model description excerpted below from the statistical analysis subsection

We used a Cox proportional hazards regression model to estimate adjusted associations between time-varying predictors of interest, including time-since-vaccination status, COVID exposure, reason for testing, and symptoms, and risk of positive SARS-COV-2 test result. The effect of predictors of interest is adjusted for a dynamic baseline risk of infection which is estimated non-parametrically, and static covariates including gender, community role defined as student, employee, or other, and age at baseline.

11. The manuscript requires a comprehensive English editing including an editing of sentence and paragraph structure.

We appreciate the suggestion and have edited the manuscript.

Reviewer: 4
Prof. Berent Prakken, UMC Utrecht Faculty of Medicine

Comments to the Author:
In this prospective cohort study Robinson et al systematically tracked symptoms from a cohort of individuals at Drexel University March 2020 to April 2022, so at the heart of the COVID pandemic. They used these data on vaccination, SARS-COV-2 PCR testing to assess

the association between among others vaccination status, symptoms and the risk of a positive test result. This was done in a prospective cohort 15.527 University students, faculty, and staff (n=15,527) at Drexel that underwent mandatory COVID-19 symptom tracking, testing and vaccinations.
I think that there are various important novelties in this study that adds to already extensive wealth of data on the dynamics of the COVID pandemic. First of all the large prospective cohort albeit with a relatively young population. Next the use of a mobile device to track the use of an application on mobile devices for tracking the symptoms.
This is as far as I know a quite unique, real-world study that gives important new insights in the dynamics of the COVID pandemic in a well-described cohort. It provides a fascinating picture of the first important two years of the COVID pandemic and the efficacy of vaccination on both symptomatic and asymptomatic infections. One of the most relevant findings is that the overall effect of vaccination on asymptomatic infections was quite low (estimated overall effect on infections within 90 days around 37,3%). Their overall conclusion, namely that the differences in vaccine effectiveness over time appear to be driven primarily by evolution of individual level time-since vaccine status and not so much by changes in the virus, is in my opinion very significant. Given the way the study was structured, the use of the mobile app and the impressive amount of data makes this a robust study that in my opinion yields important new insights in the dynamics of the COVID pandemic. It would even be advisable to add a commentary or editorial to put this study in a broader perspective.

Minor remarks: some small typo's in the manuscript (eg requited instead of required)

We appreciate the reviewer's response to the novelty and relevance of our study, and are considering how these findings could be incorporated into a broader statement about vaccine effectiveness and COVID control policies.
